# Prevalence, Recognition and Correlates of Obsessive–Compulsive Symptoms in a Clozapine-Treated Cohort: A Service Evaluation From Suffolk.

**DOI:** 10.1192/bjo.2026.11615

**Published:** 2026-06-30

**Authors:** Nada Zahreddine, Lucy Mountford, Kim Newton, Amanda Mackay, Mohammed Miah, Mohamed Elkoshiery, Maria Giner-Murillo, Ahmed Ibrahim, Angelos Zikos

**Affiliations:** Norfolk and Suffolk NHS Foundation Trust, Ipswich, United Kingdom; Mohamed.elkoshiery@nsft.nhs.uk, ST, Maria.giner-murillo@nsft.nhs.uk, CT, Ahmed.ibrahim@nsft.nhs.uk, CT, Angelos.zikos@nsft.nhs.uk, CT, All at NSFT, Ipswich, UK]

## Abstract

**Aims:**

Obsessive–compulsive symptoms (OCS) are a reported complication of clozapine treatment but remain under-recognised. Real-world data on their prevalence and clinical correlates in the UK remain limited.

**Methods:**

This cross-sectional service evaluation was conducted within a centralised clozapine clinic serving adult secondary care services across Suffolk. Patients receiving clozapine were invited to complete the Obsessive–Compulsive Inventory–Revised (OCI-R) as part of routine clinical assessment. Demographic, clinical, and service-use data were extracted from electronic health records. Illness severity and symptom domains were assessed using the Clinical Global Impression–Schizophrenia scale (CGI-SCH), and functioning using the Global Assessment of Functioning (GAF).

**Results:**

A total of 187 clozapine-treated patients (mean age 46.6 years, SD=12.4; 70.1% male) completed the OCI-R, which showed good internal consistency (α=.87); the mean OCI-R OCD score was 13.2 (SD=9.8).

Overall, 52.4% met criteria for OCS (OCI-R OCD ≥ 12), yet only 8.6% had OCS documented by a psychiatrist in the preceding 18 months, with poor agreement between measures (p < .001), indicating substantial under-recognition. Among patients meeting OCI-R criteria, overall OCS severity did not differ between recognised and unrecognised cases (p=.153); however, clinician recognition was uniquely associated with higher checking subscale scores (p=.004), suggesting preferential identification of more overt compulsive behaviours.

Multivariable linear regression predicting OCI-R OCD scores was statistically significant (p < .001), with higher CGI-SCH positive and cognitive symptom subscale scores independently predicting greater OCS severity after adjustment for demographic, clinical, and treatment-related variables, suggesting that OCS may cluster with a more severe psychosis phenotype and/or shared cognitive-control deficits.

To examine whether positive symptoms inflated OCS scores via overlap with obsessing items, a separate regression predicting the OCI-R obsessing subscale was conducted (p < .001). Higher obsessing scores were independently associated with CGI-SCH positive (p < .001) and depressive symptom severity (p=.019), indicating that OCS severity cannot be explained solely by overlap with positive psychotic symptoms.

OCI-R hoarding subscale scores were modestly associated with CGI-SCH cognitive symptoms (r=.21, p=.005), with no other significant associations, consistent with literature linking hoarding behaviours to cognitive and executive dysfunction rather than psychotic or affective pathology.

**Conclusion:**

OCS were common yet substantially under-recognised in clozapine-treated patients. Their severity was independently associated with positive and cognitive psychotic symptom domains, supporting a clinically meaningful schizo-obsessive phenotype and highlighting the need for routine screening and proactive monitoring within clozapine services.

